# Prevalence, Comorbidity, and Correlates of Psychiatric and Substance Use Disorders and Associations with HIV Risk Behaviors in a Multisite Cohort of Women Living with HIV

**DOI:** 10.1007/s10461-018-2051-3

**Published:** 2018-02-19

**Authors:** Judith A. Cook, Jane K. Burke-Miller, Pamela J. Steigman, Rebecca M. Schwartz, Nancy A. Hessol, Joel Milam, Daniel J. Merenstein, Kathryn Anastos, Elizabeth T. Golub, Mardge H. Cohen

**Affiliations:** 1 Department of Psychiatry, University of Illinois at Chicago, 1601 West Taylor Street, 4th Floor, M/C 912, Chicago, IL 60612 USA; 2 Department of Occupational Medicine, Epidemiology, and Prevention, Hofstra Northwell School of Medicine, Great Neck, NY USA; 3 Department of Clinical Pharmacy, University of California, San Francisco, San Francisco, CA USA; 4 Institute for Health Promotion and Disease Prevention Research, University of Southern California, Los Angeles, CA USA; 5 Department of Family Medicine, Georgetown University, Washington, DC USA; 6 Department of Medicine, Albert Einstein College of Medicine, Bronx, NY USA; 7 Department of Epidemiology, Johns Hopkins University, Baltimore, MD USA; 8 Department of Medicine, Cook County Hospital Health and Hospital System, Chicago, IL USA

**Keywords:** Psychiatric epidemiology, Women living with HIV, Mental illness, Substance use disorder, Prevalence of behavioral health disorder

## Abstract

We used the World Health Organization’s Composite International Diagnostic Interview to determine the prevalence, comorbidity, and correlates of lifetime and 12-month behavioral health disorders in a multisite cohort of 1027 women living with HIV in the United States. Most (82.6%) had one or more lifetime disorders including 34.2% with mood disorders, 61.6% with anxiety disorders, and 58.3% with substance use disorders. Over half (53.9%) had at least one 12-month disorder, including 22.1% with mood disorders, 45.4% with anxiety disorders, and 11.1% with substance use disorders. Behavioral health disorder onset preceded HIV diagnosis by an average of 19 years. In multivariable models, likelihood of disorders was associated with women’s race/ethnicity, employment status, and income. Women with 12-month behavioral health disorders were significantly more likely than their counterparts to engage in subsequent sexual and substance use HIV risk behaviors. We discuss the complex physical and behavioral health needs of women living with HIV.

## Resumen

Utilizamos la Entrevista Diagnóstica Internacional Compuesta de la Organización Mundial de la Salud para determinar la prevalencia, la comorbilidad y los correlatos de trastornos de salud conductual de la vida y de 12 meses en una cohorte multisitio de 1.027 mujeres que viven con VIH en los Estados Unidos. La mayoría (82.6%) tuvo uno o más trastornos en su vida, incluyendo 34.2% con trastornos del estado de ánimo, 61.6% con trastornos de ansiedad y 58.3% con trastornos por el uso de las sustancias. Más de la mitad (53.9%) tuvo al menos un trastorno en los últimos 12 meses, incluyendo 22.1% con trastornos del estado de ánimo, 45.4% con trastornos de ansiedad y 11.1% con trastornos por el uso de las sustancias. El inicio del trastorno de salud conductual precedió el diagnóstico de VIH en un promedio de 19 años. En modelos multivariables, la probabilidad de trastornos se asoció con la raza/etnia de las mujeres, el estado de empleo y los ingresos. Las mujeres con trastornos de salud conductual de 12 meses fueron considerablemente más probable que sus homólogas a participar en comportamientos de riesgo de VIH como el uso de las sustancias y conductas sexuales de riesgo. Discutimos las necesidades complejas de salud física y conductual de las mujeres que viven con el VIH.

## Introduction

In the US, the prevalence of psychiatric disorders and substance use disorders among people living with HIV is thought to be high [1–3]. However, estimates typically are derived from small, non-representative samples because population-based studies are rare, and most research uses screening rather than full diagnostic assessment. Moreover, prior research has addressed a limited range of specific disorders or diagnostic groupings, with few reports on a comprehensive array of psychiatric disorders and substance use disorders. The purpose of this study was to determine the prevalence, comorbidity and correlates of a full range of behavioral health disorders in a longitudinal multisite cohort of women living with HIV participating in the Women’s Interagency HIV Study or WIHS.

To date, the sole nationally representative study of behavioral health disorders among HIV-positive US adults is the 1996–1997 HIV Cost and Services Utilization Study (HCSUS) [4]. Using the Composite International Diagnostic Interview (CIDI) Short Form, HCSUS participants were screened for major depression, dysthymia, generalized anxiety disorder, and panic disorders in the past year [5]. Close to half screened positive for one or more of these psychiatric disorders (47.9% of the weighted sample). The full CIDI diagnostic interview was administered separately to a subsample of the original cohort, and results were used to refine prevalence estimates [6] resulting in a 32.8% prevalence of any 12-month psychiatric disorder: 22.0% for major depression; 5.0% for dysthymia; 4.1% for generalized anxiety disorder; and 15.5% for panic disorder. While lower than those obtained by screening, these estimates were considerably higher than those found in a survey of the general population which used the full CIDI and found prevalence rates of 26.2% for any psychiatric disorder, 6.7% for major depression, 1.5% dysthymia, 3.1% generalized anxiety disorder, and 2.7% panic disorder [7, 8].

Clinical studies of smaller, non-representative samples report the prevalence for any current psychiatric disorder of 33–63% among HIV-positive adults [9–13]. Research on depressive disorders finds prevalence estimates ranging from 10 to 20% [11, 14]. Anxiety disorders also are prevalent, with estimates ranging from 5 to 15%, with the most common being generalized anxiety disorder and social anxiety disorder [9, 14, 15].

Information about the prevalence of substance use disorders among HIV-positive individuals is also lacking [1–3]. In the HCSUS CIDI-Short Form screening study, 50.1% of participants reported illicit drug use in the past year and 12% met criteria for drug dependence [5]. Close to a fifth (18.5%) met criteria for alcohol use disorders [4].

High rates of co-occurring psychiatric disorders and substance use disorders are reported among people living with HIV [10, 16, 17]. In the HCSUS, the 12-month prevalence of co-occurring disorders was 10% [18, 19]. Also in the HCSUS, multivariable analysis revealed that the presence of any psychiatric disorder was significantly associated with drug dependence, and vice versa [5]. However, there is very little information on co-occurrence of substance use disorders with specific psychiatric syndromes, which inhibits our understanding of how these conditions interact, and their impact on HIV transmission behaviors and outcomes [20].

Turning next to HIV-positive women, a longitudinal multisite study of 1223 HIV-infected birth mothers found that 32% screened positive for psychiatric disorder, 9% for substance use disorder, and 35% for psychiatric disorder and/or substance use disorder [21]. Another study examined electronic medical records of HIV-positive patients in California, including 738 women, in which psychiatric disorder and substance use disorder diagnoses were based on clinician judgment and patient self-report [22]. In this study, 50% of female patients had a documented psychiatric disorder, including 25% with a mood disorder, 16% with an anxiety disorder and 12% with a substance use disorder. Among 1710 participants in the longitudinal multisite HIV-positive WIHS cohort, 55% met criteria for probable depression [23] and they also reported high rates of alcohol [24] and substance use [25]. These comorbidities often occur in a context of poverty, trauma, housing instability, caregiving demands, racial/ethnic discrimination, and multiple health needs [26–28].

A number of studies have documented associations between behavioral health disorders and engaging in HIV transmission behaviors [29, 30]. These include sexual risk behaviors such as unprotected sex [31], having multiple sex partners [32], and receptive anal intercourse [33]. Also included are drug-related risk behaviors such as sharing needles [34], and exchanging sex for drugs, money or other material items [34, 35]. An even greater likelihood of HIV risk behaviors has been observed among people with co-occurring substance use disorders and psychiatric disorders [36–38], including major depressive disorder [39] and post-traumatic stress disorder [40]. Moreover, while awareness of one’s HIV positive serostatus appears to decrease the likelihood of engaging in risk behaviors [41] even among those with psychiatric and/or substance use disorders [42], a noteworthy proportion of individuals living with HIV continue to engage in high-risk sex and drug use behaviors [43], creating opportunities for viral transmission to uninfected individuals [44].

Given the foregoing research findings and the remaining gaps in our knowledge, the present study addressed three major research questions. First, what are the lifetime and 12-month prevalence and severity of a full range of *DSM*-*IV* disorders in a multisite study cohort of women living with HIV, and what syndromes are most frequently comorbid? Second, what are the individual correlates associated with psychiatric disorders and substance use disorders among these women? And third, are specific behavioral health disorders, including co-occurring conditions, associated with a greater likelihood of engaging in HIV risk behaviors, controlling for confounding factors?

## Methods

### Study Participants

The WIHS is a multisite cohort study of women living with or at risk of acquiring HIV infection recruited in the mid-1990s at six US sites: Brooklyn, Bronx, Chicago, Los Angeles, San Francisco/Bay Area, and Washington, DC. Details of the WIHS cohort and study design can be found in prior publications [45, 46].

For this nested study, inclusion criteria were being an active HIV-positive WIHS participant, able to understand spoken English, and capable of completing an in-person assessment. From 2010 through 2013, 1213 English-speaking women were approached and 1033 consented to participate (85.2%), providing written informed consent using procedures approved by the University of Illinois at Chicago (UIC) Institutional Review Board (IRB), and the IRBs at each study site. Data for this analysis include 1027 women, representing 74.2% of the 1385 who were active as of 2010. The remaining 358 women were not included for the following reasons: unfinished interviews (n = 6); active refusals (n = 60); passive refusals whom we were unable to locate or schedule (n = 126); and exclusively Spanish-language speakers (n = 166). Comparisons between the 1027 study participants and the remaining 358 found no significant differences in age, education, income, marital status, CD4 + cell count, HIV-1 viral load, or use of combination antiretroviral therapy (cART). However, compared to the remaining 358 non-participants, a larger proportion of study completers were African American (78.4% vs. 64.4% of non-completers, Chi square = 13.91, p < 0.001), and not employed (68.2% vs. 55.5% of non-completers, Chi square = 11.71, p = 0.001). At all but one site, response rates ranged from 71 to 89%, with the remaining site (Los Angeles) having a low response rate of 40% due to the high concentration of Spanish-only speakers.

### Measures

WIHS research interviewers from each site completed 3 days of training on the use of the World Health Organization *CIDI 3.0 Auto Version*, a comprehensive, fully standardized battery that assessed disorders according to the criteria of the *DSM*-*IV,* hereafter referred to as the WMH-CIDI [47]. Training was provided by either the University of Michigan’s WHO-designated WMH-CIDI training center or by WHO-trained UIC research staff. Interviews were administered via laptop software programmed with skip patterns, error screening, and consistency checks. A randomly selected 5% of respondents were contacted via telephone by the UIC study manager following the interview, to verify interview process and completion.

Participant age, race/ethnicity, education, employment, income, marital status, study site, and CD4+ cell count were ascertained from the WIHS semiannual study visit closest to the WMH-CIDI assessment. Participant risk behaviors were assessed at up to 6 semiannual visits following the WMH-CIDI assessment. HIV sexual risk behavior was defined as exchanging sex for money/drugs/shelter, multiple current male partners, or receptive anal sex; and substance use risk behavior was defined as use of any illicit drug or high risk drinking defined as consumption of > 7 standard alcoholic drinks per week in accordance with the National Institute on Alcohol Abuse and Alcoholism [48].

### Statistical Analysis

Frequency distributions and cross-tabulations were used to calculate prevalence, severity, and comorbidity of behavioral health diagnoses in the WIHS cohort and the 2003 National Comorbidity Survey Replication (NCS-R) women’s cohort. Prevalence and standard errors in the NCS-R data were calculated using the study documentation manual’s survey strata, clusters, and weights [49]. Adjusted logistic regression analysis examined associations between participant characteristics and behavioral health disorders. Random effects logistic regression models (RRM) analyzed the prospective relationships between 12-month diagnoses and HIV sexual and substance use risk behaviors. Analyses were conducted in IBM SPSS Statistics 23 and SAS 9.4.

## Results

### Lifetime *DSM*-*IV* Disorders and Age of Onset

Characteristics of the study participants are shown in Table 1. Table 2 presents the lifetime prevalence of *DSM*-*IV* disorders in our study population and compares it to that of the US adult women’s general household population in the NCS-R. As shown at the bottom of column 1, the majority of our study population (82.6%) reported a lifetime *DSM*-*IV* disorder versus just under half of the general US women’s population (45.7%) shown in column 2. Prevalence of lifetime mood disorders in the study population was considerably higher than that of the general population (34.2% vs 23.7%) as were anxiety disorders (61.6% vs 37.0%), and substance use disorders, which were 6 times more likely in the study group versus the general population (58.3% vs 8.8%). The most common specific lifetime disorders were drug abuse (49.4%), major depressive disorder (32.4%), and posttraumatic stress disorder (PTSD) (29.8%).

**Table 1:** Demographic and clinical characteristics of study population (n = 1027)

	n = 1027 Mean (SD) or %(n)
Age, years	48.1 (8.7)
Age, quartiles (%)	
27–39 years	16.9 (174)
40–49 years	40.1 (412)
50–59 years	34.5 (354)
60 + years	8.5 (87)
Race (%)	
White	17.1 (176)
Black	78.4 (805)
Asian	1.0 (10)
Native American	0.4 (4)
Other	3.1 (32)
Hispanic Ethnicity (%)	18.8 (193)
Education completed (%)	
0–11 years	33.1 (339)
12 years	31.9 (327)
13–15 years	26.9 276)
16 + years	8.1 (84)
Employed full or part-time (%)	31.8 (327)
Annual household income ≤ $12,000 (%)	48.7 (466)
Marital Status (%)	
Married/cohabiting	36.9 (379)
Previously married	31.1 (319)
Never married	32.0 (329)
Site (%)	
Brooklyn, NY	25.2 (259)
Bronx, NY	17.3 (178)
Chicago, IL	14.2 (146)
Los Angeles, CA	11.7 (120)
San Francisco, CA	15.0 (154)
Washington, DC	16.6 (170)
Taking combination antiretroviral therapy (cART) (%)	87.0 (893)
CD4 cell count (%)	
< 200 mm^3^	11.8 (121)
200+ mm^3^	88.2 (906)
HIV RNA viral load (%)	
> 200 copies/mL	28.8 (295)
≤ 200 copies/mL	71.2 (731)
Engaged in HIV risk behaviors over 3 years post-*DSM*-*IV* diagnostic assessment (%)	
Risky sexual behaviors	16.2 (166)
Risky substance use behaviors	38.1 (390)

*SD* standard deviation, *DSM*-*IV* diagnostic and statistical manual of mental disorders, 4th edition

**Table 2:** Lifetime prevalence of *DSM-IV* disorders in the study population and general US women’s population, and age of onset of *DSM-IV* disorders and HIV diagnosis in the study population (N = 1027)

*DSM*-*IV* diagnostic category	Study population prevalence % (SE)	General US women’s population^a^ prevalence % [SE)	Average age of onset, in years	Average age of HIV diagnosis, in years	Average # years disorder preceded HIV diagnosis (95.5%)	Average # years HIV diagnosis preceded disorder (4.5%)
Mood disorders						
Major depressive disorder	32.4 (1.5)	22.9 (0.6)	20.04	32.35	15.94	8.62
Dysthymia	9.9 (0.9)	5.1 (0.4)	19.52	32.18	16.66	6.28
Bipolar I and II disorders	6.6 (0.8)	2.4 (0.3)	24.28	32.16	13.91	9.08
Any mood disorder	34.2 (1.5)	23.7 (0.6)	20.19	32.46	15.83	8.68
Anxiety disorders						
Panic disorder	9.0 (8.9)	6.2 (0.3)	23.83	31.72	14.76	9.75
Agoraphobia	6.2 (0.7)	2.9 (0.3)	16.80	32.50	20.16	8.89
Specific phobia	28.0 (1.4)	15.8 (0.6)	9.54	32.34	24.11	8.83
Social phobia	19.7 (1.2)	13.0 (0.6)	13.65	33.34	21.34	8.76
Generalized anxiety disorder	13.2 (1.0)	9.9 (0.4)	24.44	33.13	16.52	10.11
Posttraumatic stress disorder	29.8 (1.4)	9.7 (0.7)	23.68	32.29	14.53	8.38
Obsessive–compulsive disorder	16.0 (1.1)	3.1 (0.5)^b^	20.02	31.69	17.92	8.66
Separation disorder (as child)	10.7 (1.0)	5.4 (0.5)	8.44	32.94	24.51	N/A
Separation disorder (as adult)	15.9 (1.1)	7.4 (0.3)	26.78	31.36	12.74	8.60
Any anxiety disorder	61.6 (1.5)	37.0 (1.2)	13.19	32.64	22.11	9.47
Substance use disorders						
Alcohol w/wo dependence	19.6 (1.2)	7.5 (0.5)	22.88	33.47	13.88	8.27
Drug abuse w/wo dependence	49.4 (1.6)	4.8 (0.4)	20.43	33.52	14.07	5.49
Any substance use disorder	58.3 (1.5)	8.8 (0.5)	20.99	33.54	14.03	6.80
Any *DSM*-*IV* disorder	82.6 (1.2)	45.7 (1.1)	13.96	33.01	20.20	6.49

*SE* standard error, *w/wo* with or without, *DSM*-*IV* diagnostic and statistical manual of mental disorders, 4th edition

^a^National Comorbidity Survey Replication (NCS-R) [49]

^b^From published NCS-R results. This disorder is not available in NCS-R data and is therefore excluded from summary prevalence calculations (e.g., any mood, anxiety, substance use or *DSM*-*IV* disorders)

The average age of onset for different *DSM*-*IV* disorders is presented in column 3 of Table 2. The vast majority of mood disorders, anxiety disorders, and substance use disorders had an average age of onset in the early- to mid-20s. The exceptions to this are earlier onset for several anxiety disorders including specific phobia (9.5 years of age), social phobia (13.6 years), and agoraphobia (16.8 years). Also, by definition, childhood separation anxiety disorder has an average age of onset in childhood, at 8.4 years.

The average age of HIV diagnosis for those with particular disorders is presented in column 4. Regardless of type of psychiatric disorder or substance use disorder, the average age at HIV diagnosis was in the early 30s, with the average age being earliest for those with panic disorder (31.7 years), obsessive–compulsive disorder (OCD) (31.7 years), and adult separation anxiety disorder (31.4 years), and latest for social phobia (33.3 years), generalized anxiety disorder (33.1), and all of the substance use disorders (all > 33.3 years of age).

For the large majority of participants (95.5%) *DSM*-*IV* disorder onset preceded HIV diagnosis, and column 5 presents the average number of years between disorder onset and HIV diagnosis for this group. On average, mood disorder onset was 15.83 years prior to HIV diagnosis; anxiety disorder onset was 22.11 years prior to HIV diagnosis; and substance use disorder onset pre-dated HIV diagnosis by 14.03 years. Among the minority of women (4.5%) whose HIV diagnosis predated their *DSM*-*IV* disorder onset, as shown in column 6, onset for mood disorders was an average of 8.68 years after HIV diagnosis; onset for anxiety disorders was 9.47 years after HIV diagnosis; and onset of substance use disorders occurred earliest, an average of 6.80 years after HIV diagnosis.

### 12-Month *DSM*-*IV* Disorders and Severity

Turning next to 12-month *DSM*-*IV* disorders, Table 3 presents their prevalence and severity in the WIHS cohort and corresponding information regarding the US women’s general household population in the NCS-R. Mood disorder prevalence in the WIHS cohort was 22.1% compared to 10.8% in the general women’s population. Anxiety disorder prevalence was 45.4% in the WIHS compared to 23.2% in the general population, substance use disorders were 5 times as prevalent in the WIHS compared to the general women’s population (11.1% vs. 2.2%). The most common mood disorder was major depressive disorder (20.0%). The most common anxiety disorders were specific phobias (22.1%), PTSD (16.6%), and social phobia (13.9%). Among substance use disorders, drug abuse with or without dependence (5.0%) was more common than alcohol abuse with or without dependence (2.5%). Specific disorders with noticeably higher prevalence (i.e., 3 or more times) among women living with HIV than the general women’s population were dysthymia, bipolar disorders, agoraphobia, OCD, PTSD, and drug abuse.

**Table 3:** 12-Month prevalence of *DSM-IV* disorders in the study population and general United States women’s population, and *DSM* disorder severity in the study population (n = 1027)

*DSM*-*IV* diagnostic category	Study population prevalence n = 1027 % (SE)	General US women’s population^a^ prevalence % (SE)	Severity^b^ of disorder in study population^c^ n = 1027		
			Serious % (SE)	Moderate % (SE)	Mild % (SE)
Mood disorders					
Major depressive disorder	20.0 (1.2)	10.2 (0.5)	80.0 (2.8)	14.6 (2.5)	5.4 (1.6)
Dysthymia	9.9 (0.9)	2.9 (0.2)	88.2 (3.2)	7.9 (2.7)	4.0 (1.9)
Bipolar I and II disorders	5.2 (0.7)	1.5 (0.2)	60.4 (6.8)	15.1 (5.0)	24.5 (6.0)
Any mood disorder	22.1 (1.3)	10.8 (0.5)	81.1 (2.6)	14.1 (2.3)	4.8 (1.4)
Anxiety disorders					
Panic disorder	5.8 (0.7)	3.8 (0.3)	73.3 (5.7)	20.0 (5.2)	6.7 (3.2)
Agoraphobia	4.8 (0.7)	1.6 (0.2)	63.3 (6.9)	14.3 (5.1)	22.4 (6.0)
Specific phobia	22.1 (1.3)	11.7 (0.5)	19.4 (2.6)	42.3 (3.3)	38.3 (3.2)
Social phobia	13.9 (1.1)	7.8 (0.5)	60.8 (4.1)	18.2 (3.2)	21.0 (3.4)
Generalized anxiety disorder	7.9 (0.8)	5.2 (0.3)	65.4 (5.3)	13.6 (3.8)	21.0 (4.5)
Posttraumatic stress disorder	16.6 (1.2)	5.2 (0.4)	47.4 (3.8)	18.1 (3.0)	34.5 (3.6)
Obsessive–compulsive dis.	8.1 (0.8)	1.8 (0.5)^d^	54.2 (5.5)	14.5 (3.9)	31.3 (5.1)
Separation disorder (child)	1.5 (0.4)	N/A	66.7 (12.6)	13.3 (9.1)	20.0 (10.7)
Separation disorder (adult)	5.8 (0.1)	2.1 (0.2)	70.0 (5.6)	13.3 (4.4)	16.7 ((4.8)
Any anxiety disorder	45.4 (1.6)	23.2 (0.8)	51.1 (2.3)	19.5 (1.8)	29.4 (2.1)
Substance disorders					
Alcohol abuse w/wo dep	2.5 (0.5)	1.8 (0.3)	68.0 (9.5)	28.0 (9.2)	4.0 (4.0)
Drug abuse w/wo dep	5.0 (0.7)	0.7 (0.1)	66.7 (6.7)	15.7 (5.1)	17.6 (5.4)
Any substance disorder	11.1 (1.0)	2.2 (0.3)	47.4 (4.7)	19.3 (3.7)	33.3 (4.4)
# of *DSM*-*IV* disorders					
1 disorder	20.2 (1.2)	15.1 (0.7)	26.4 (3.1)	18.8 (2.7)	54.8 (3.4)
2 disorders	11.1 (1.0)	6.1 (0.4)	58.8 (4.6)	26.3 (4.1)	14.9 (3.4)
3 + disorders	22.6 (1.3)	6.6 (0.4)	84.9 (2.3)	11.6 (2.1)	3.4 (1.2)
Any *DSM*-*IV* disorder	53.9 (1.6)	27.8 (0.9)	57.6 (2.1)	17.2 (1.6)	25.1 (1.8)

*SE* standard error, *N/A* not available, *w/wo* with or without, *DSM*-*IV* diagnostic and statistical manual of mental disorders, 4th edition

^a^National Comorbidity Survey Replication (NCS-R) [49]

^b^Severity defined by: work disability, substantial role limitation or impairment, serious violence, or serious suicide attempt

^c^Percentages in the three severity columns are reported as proportions of all cases and sum to 100% across each row

^d^From published NCS-R results. This disorder is not available in NCS-R data and is therefore excluded from summary prevalence calculations (e.g., any mood, anxiety, substance use or *DSM*-*IV* disorders)

Among women with 12-month disorders (bottom row of Table 3 columns 3–5), over half (57.6%) had at least one classified as serious, 17.2% as moderate, and 25.1% as mild in terms of highest severity level. Mood disorders had the highest percentage of serious classifications (81.1% of women with any mood disorder had at least one serious mood disorder), followed by anxiety disorders (51.1%), and substance use disorders (47.4%). Again in column 3, the specific mood disorder with the highest percentage of serious classifications was dysthymia (88.2%); the specific anxiety disorder with the highest percentage of serious classifications was panic disorder (73.3%); and the substance use disorder with the highest percentage of serious classifications was alcohol abuse (68.0%).

Regarding number of 12-month disorders (Table 3), over two-fifths (22.6%) of WIHS study participants had three or more disorders, compared to only 6.6% in the general women’s population; 11.1% of WIHS participants had 2 disorders, compared to 6.1% in the general population, and 20.2% had one disorder compared to 15.1% in the general population. In addition, as shown in the 3rd column, there was a positive relationship between severity and multiple disorders. Here, among women with three or more disorders, 84.9% had at least one disorder classified as serious on the severity scale, 58.8% of women with two disorders had at least one serious disorder, and 26.4% of women with only 1 disorder had a serious disorder.

### Comorbidity of Lifetime and 12-Month Disorders

Comorbid disorders were common (not shown) for both lifetime and 12-month disorders. Lifetime mood disorders had the highest rate of comorbidity. Here, the large majority of women (87.2%) with a lifetime mood disorder also had a lifetime anxiety disorder, and over two-thirds (68.4%) of those with a lifetime mood disorder also had a lifetime substance use disorder. In contrast, less than half of women with lifetime anxiety disorders or substance use disorders had co-occurring lifetime mood disorders (48.3% and 40.1% respectively). In addition, lifetime anxiety disorders and substance use disorders were often comorbid, with 65.9% and 69.6% of each comorbid with the other, respectively. Similarly, 12-month mood disorders had the highest rate of comorbidity. Here, 79.7% of those with 12-month mood disorders also had 12-month anxiety disorders. However, 12-month mood disorders were less frequently comorbid with 12-month substance use disorders (20.3%) given the relatively low prevalence of 12-month substance use disorders. Similar to lifetime rates, 38.8% of those with 12-month anxiety disorders also had co-occurring 12-month mood disorders, and 40.4% of those with 12-month substance use disorders also had 12-month mood disorders. The frequent co-occurrence of 12-month anxiety disorders and substance use disorders also was observed, since 61.4% of those with 12-month substance use disorders had comorbid 12-month anxiety disorders.

### Individual Correlates of Lifetime and 12-Month Disorders

In multivariable logistic regression analysis (Table 4) with all individual characteristics entered (age, race/ethnic group, education, employment, income, marital/cohabiting status, CD4+ cell count) and controlling for study site, lifetime mood disorder was less likely among women who were working than not working [odds ratio (95% confidence interval) OR 0.7 (0.5–1.0)]. Lifetime anxiety disorder also was less likely among women who were working than not working [OR 0.6 (0.4–0.8)], and more likely among women with CD4 cell counts higher than 200 [OR 1.5 (1.0–2.3)]. Lifetime substance use disorder was significantly more likely among women 40 years or older compared to those under age 40 [age 40–49 years OR 2.2 (1.5–3.3); age 50–59 OR 4.1 (2.6–6.3); age 60 + OR 2.0 (1.1–3.7)]. Lifetime substance use disorder was less likely among Hispanic women [OR 0.4 (0.4–0.7)] and women in Other race/ethnic groups than White women [OR 0.4 (0.2–0.9)]; less likely among women with highest levels of education compared to those with the least education [16 + years OR 0.4 (0.2–0.8)]; less likely among women who were working compared to those not working [OR 0.7 (0.5–0.9)]; and less likely among women with higher income > = $12,000 than lower income [OR 0.7 (0.5–0.9)].

**Table 4:** Multivariable sociodemographic and clinical correlates of lifetime and 12-month *DSM-IV* diagnostic categories, controlling for site (N = 1027)

Sociodemographic correlates	*DSM*-*IV* cases, odds ratios (95% CI)					
	Lifetime			12-Month		
	Mood disorder OR (95% CI)	Anxiety disorder OR (95% CI)	Substance use disorder OR (95% CI)	Mood disorder OR (95% CI)	Anxiety disorder OR (95% CI)	Substance use disorder OR (95% CI)
Age, years						
27–39	1.0 (reference)	1.0 (reference)	1.0 (reference)	1.0 (reference)	1.0 (reference)	1.0 (reference)
40–49	1.4 (0.9–2.1)	1.0 (0.6–1.4)	2.2 (1.5–3.3)***	1.3 (0.8–2.2)	0.8 (0.6–1.2)	1.2 (0.6–2.4)
50–59	1.2 (0.8–1.9)	1.4 (0.9–2.1)	4.1 (2.6–6.3)***	1.3 (0.8–2.2)	1.1 (0.7–1.6)	1.2 (0.6–2.6)
60^+^	0.7 (0.4–1.4)	0.7 (0.4–1.2)	2.0 (1.1–3.7)*	0.7 (0.3–1.5)	0.4 (0.2–0.8)*	0.4 (0.1–1.4)
Race/ethnicity						
Non-Hispanic White	1.0 (reference)	1.0 (reference)	1.0 (reference)	1.0 (reference)	1.0 (reference)	1.0 (reference)
Non-Hispanic Black	0.7 (0.5–1.1)	1.0 (0.7–1.6)	0.7 (0.4–1.1)^+^	0.5 (0.3–0.9)*	1.0 (0.7–1.5)	0.5 (0.3–0.9)*
Hispanic	1.1 (0.6–1.8)	1.5 (0.9–2.4)	0.4 (0.2–0.7)**	0.5 (0.3–0.9)*	1.2 (0.8–2.0)	0.4 (0.2–0.8)*
Other	1.2 (0.6–2.7)	0.5 (0.2–1.1)^+^	0.4 (0.2–0.9)*	0.8 (0.3–2.0)	0.7 (0.3–1.5)	0.4 (0.1–1.6)
Education completed (years)						
0–11	1.0 (reference)	1.0 (reference)	1.0 (reference)	1.0 (reference)	1.0 (reference)	1.0 (reference)
12	1.0 (0.7–1.4)	0.8 (0.6–1.1)	0.9 (0.6–1.3)	1.0 (0.7–1.5)	0.7 (0.5–0.9)*	0.8 (0.5–1.5)
13–15	1.3 (0.9–1.8)	0.9 (0.6–1.2)	0.8 (0.6–1.2)	1.1 (0.7–1.7)	0.8 (0.6–1.1)	1.0 (0.5–1.7)
16^+^	0.9 (0.5–1.7)	1.0 (0.6–1.7)	0.4 (0.2–0.8)**	0.8 (0.4–1.7)	0.8 (0.5–1.4)	0.7 (0.2–1.9)
Work status						
Not working	1.0 (reference)	1.0 (reference)	1.0 (reference)	1.0 (reference)	1.0 (reference)	1.0 (reference)
Working (part or full-time)	0.7 (0.5–1.0)*	0.6 (0.4–0.8)**	0.7 (0.5–0.9)*	0.4 (0.2–0.6)***	0.5 (0.4–0.7)***	0.6 (0.3–1.1)^+^
Income						
≤ 12,000	1.0 (reference)	1.0 (reference)	1.0 (reference)	1.0 (reference)	1.0 (reference)	1.0 (reference)
> 12,000	0.8 (0.6–1.1)	0.9 (0.6–1.2)	.07 (0.5–0.9)*	0.6 (0.4–0.9)*	0.9 (0.6–1.2)	0.4 (0.2–0.6)***
Marital status						
Married/cohabiting	1.0 (reference)	1.0 (reference)	1.0 (reference)	1.0 (reference)	1.0 (reference)	1.0 (reference)
Previously married	1.0 (0.7–1.4)	1.3 (0.9–1.8)	1.0 (0.7–1.4)	1.1 (0.7–1.6)	1.4 (1.0–1.9)^+^	0.7 (0.4–1.2)
Never married	1.0 (0.7–1.4)	1.3 (0.9–1.8)	1.1 (0.8–1.6)	1.2 (0.8–1.8)	1.2 (0.9–1.7)	1.4 (0.8–2.3)
CD4 cell count, %						
< 200	1.0 (reference)	1.0 (reference)	1.0 (reference)	1.0 (reference)	1.0 (reference)	1.0 (reference)
200^+^	1.4 (0.9–2.2)	1.5 (1.0–2.3)*	1.1 (0.7–1.7)	1.2 (0.7–1.9)	1.5 (1.0–2.2)^+^	0.7 (0.4–1.2)

*OR* odds ratio, *CI* confidence interval

^+^*p* < 0.10, *p < 0.05, **p < 0.01, ***p < 0.001

Models adjusted for study site

*DSM*-*IV* diagnostic and statistical manual of mental disorders, 4th edition

Also in Table 4, 12-month mood disorder was less likely among Black women [OR 0.5 (0.3-0.9)] and Hispanic/Latina women [OR 0.5 (0.3–0.9)]; less likely among employed women than those not working [OR 0.4 (0.2–0.6)]; and less likely among women with higher income (i.e., > = $12,000) than lower income [OR 0.6 (0.4–0.9)]. Anxiety disorder was less likely among women 60 + years old compared to those < 40 years old [OR 0.4 (0.2–0.8)]; less likely among those with a high school or equivalent education compared to those with less education [OR 0.7 (0.5–0.9)]; and less likely among those who were working compared to not working [OR 0.5 (0.4–0.7)]. Substance use disorders were less likely among Black women [OR 0.5 (0.3–0.9)] and Hispanic/Latina women [OR 0.4 (0.2–0.8)], and less likely among women with higher income [OR 0.4 (0.2–0.6)] than lower income.

### Association of 12-Month Disorders with Subsequent HIV Risk Behaviors

Our final research question was whether HIV-positive women with *DSM*-*IV* disorders were more likely to engage in HIV risk behaviors than those without disorders, and whether specific disorders or disorder combinations were associated with greater risk. In random effects logistic regression models adjusted for time, age, race (Latina or White vs. Black), education (< high school), and study site, we found significant associations between diagnosis of 12-month psychiatric disorders or substance use disorders, as well as their co-occurrence, and the likelihood of engaging in subsequent sexual HIV risk behaviors (Table 5). Associations were particularly strong for women with mood disorders (major depression, dysthymia, bipolar disorders) and substance use disorders (alcohol or drug abuse/dependence). For example, compared to their counterparts, women with 12-month bipolar disorders were over 13 times as likely [OR 13.6 (4.1–45.3)] and those with drug abuse disorders over 14 times as likely [OR 14.3 (4.3–47.1)] to engage in sexual risk behaviors in the ensuing 3 years. There also were significant associations with most anxiety disorders including panic, specific phobia, social phobia, PTSD, OCD, and adult separation anxiety disorder. Agoraphobia and generalized anxiety disorder were not associated with subsequent sexual risk behaviors. Women with any psychiatric disorder and co-occurring substance use disorder were over 10 times as likely to engage in risky sexual behaviors [OR 10.4 (3.7–29.7)], and women with any mood disorder and co-occurring substance use disorder were over 15 times as likely [OR 15.9 (4.4–57.9)].

**Table 5:** Results of random effects logistic regression models showing the prospective relationship of 12-month *DSM*-*IV* mood, anxiety and substance use disorders to subsequent HIV risk behaviors over 3 years (n = 1024 women, n = 4488 study visits)

12-month *DSM*-*IV* diagnostic category	Risky sexual behavior^a^ 16% (166) of women OR (95% CI)	Risky alcohol/drug use^b^ 38% (390) of women OR (95% CI)
Mood disorders		
Major depressive disorder	5.96 (2.78, 12.77)***	4.43 (1.89, 10.39)**
Dysthymia	4.19 (1.58, 11.15)**	1.79 (0.58, 5.56)
Bipolar I and II disorders	13.57 (4.06, 45.34)***	1.70 (0.39, 7.45)
Any mood disorder	5.70 (2.75, 11.86)***	4.52 (2.02, 10.12)***
Anxiety disorders		
Panic disorder	4.67 (1.40, 15.65)*	2.80 (0.67, 11.69)
Agoraphobia	1.99 (0.49, 8.17)	1.96 (0.41, 9.42)
Specific phobia	2.54 (1.20, 5.38)**	1.78 (0.78, 4.08)
Social phobia	3.74 (1.57, 8.91)***	2.14 (0.80, 5.71)
Generalized anxiety disorder	1.87 (0.57, 6.06)	3.04 (0.87, 10.62)
Posttraumatic stress disorder	2.30 (1.01, 5.20)*	1.58 (0.62, 4.02)
Obsessive–compulsive dis.	5.26 (1.88, 14.69)**	1.34 (0.38, 4.79)
Separation disorder (adult)	3.40 (1.03, 11.21)*	1.72 (0.41, 7.29)
Any anxiety disorder	4.46 (2.26, 8.78)***	2.84 (1.40, 5.76)**
Substance use disorders		
Alcohol abuse	6.20 (1.17, 32.80)***	69.05 (8.85, 538.76)***
Alcohol dependence	9.09 (1.67, 49.64)***	51.14 (6.44, 406.17)***
Drug abuse	14.28 (4.30, 47.14)***	67.93 (15.26, 302,36)***
Drug dependence	11.40 (3.79, 34.30)***	20.79 (5.62, 76.83)***
Any substance use disorder	6.60 (2.64, 16.50)***	48.53 (16.99, 138.53)***
Co-occurring psychiatric + substance use disorders		
Any mood + any substance use	15.88 (4.35, 57.92)***	23.42 (4.87, 112.58)***
Any anxiety + any substance use	9.54 (3.20, 28.44)***	16.96 (4.66, 61.68)***
Any psychiatric + any substance use	10.43 (3.67, 29.67)***	19.57 (5.68, 67.42)***

*OR* odds ratio, *CI* confidence interval, *DSM*-*IV* diagnostic and statistical manual of mental disorders, 4th edition

*p < 0.05, **p < 0.01, ***p < 0.001

Models adjusted for time, age (in 10 year increments), race (Latina or White vs. Black), education (< high school vs. high school or more) and study site (Brooklyn as reference)

Three women did not have study visits with behavioral data subsequent to the WMH-CIDI assessment

^a^Exchanged money for sex/drugs/shelter, multiple male partners, or receptive anal sex

^b^Any illicit drug use or consumption of > 7 National Institute on Alcohol Abuse and Alcoholism standard drinks per week

By definition, strong associations were expected between substance use disorders and the subsequent occurrence of HIV drug and alcohol risk behaviors, and these are evident in Table 5. However, women with any mood disorder or any anxiety disorder also were more likely to engage in HIV drug and/or alcohol risk behaviors than others [OR 4.5 (2.0–10.1), and OR 2.8 (1.4–5.8), respectively].

## Discussion

Results from this investigation confirm those of the only other large scale assessment of behavioral health disorders among adults living with HIV, which was conducted almost three decades ago. The lifetime and 12-month prevalence of behavioral health disorders in this US cohort study of HIV-positive women far exceeded that of the general population of US women, with mood disorders, anxiety disorders, and substance use disorders being two- to three-fold more common in our cohort. Also, the number of *DSM*-*IV* disorders was higher in our cohort than in the general US women’s population, with 23% having 3 or more 12-month disorders compared to only 7% in the general population. Another noteworthy difference was in the distribution of lifetime disorders, with substance use disorders being second most common in our cohort, while they were least common in the general women’s population.

This study also found a high degree of comorbidity across mood disorders, anxiety disorders, and substance use disorders. This has been noted in studies of the general population [50, 51], but the large extent to which it characterizes HIV-positive women is evident for the first time in our findings. Almost all women with lifetime mood disorders had co-occurring lifetime anxiety disorders (87%) while over two-thirds (68%) also had lifetime substance use disorders. Thus the occurrence of no behavioral health disorders or only one disorder was uncommon in this longitudinal study cohort. Among women with *current* 12-month disorders, the large majority with mood disorders also had a co-occurring anxiety disorder (80%), and of those with 12-month substance use disorders, almost two-thirds (61%) had a co-occurring anxiety disorder. This extremely high level of comorbidity requires complex treatment protocols and has negative prognostic implications for psychological health, medical well-being, and longevity [52, 53].

We found that sociodemographic characteristics including education, income, being Black, and being Latina were negatively associated with likelihood of behavioral health disorders which mirrors prior CIDI research on the general population [8]. Employed women were less likely to have a recent behavioral health disorder, which parallels the well-established negative relationship between behavioral health disorders and employment [54].

Finally, this study documented significant associations between behavioral health disorders and HIV sexual and drug use risk behaviors in the 3 years following diagnostic assessment. Risky sexual behaviors were more likely among women with any 12-month disorder, and were especially likely among women with diagnoses of bipolar disorder or drug use disorders. In addition, a 12-month diagnosis of co-occurring mood disorder with substance use disorder or co-occurring anxiety disorder with substance use disorder increased the likelihood of subsequent risky drug and alcohol use. Drug and alcohol risk behaviors can be associated with sexual risk behaviors, both because women exchange sex for material goods and because decision making capacity is diminished when under the influence. Thus, our results suggest that interventions addressing behavioral health may also prevent subsequent HIV risk behaviors. For example, it may be beneficial to tailor HIV risk reduction interventions for women with disorders in which impulse control can be problematic, such as bipolar and substance use disorders [55], since these had the highest odds ratios for HIV transmission behaviors. Similarly, interventions might effectively be tailored for women with high levels of negative affect and associated risk taking [56], such as major depression and obsessive–compulsive disorder [57], since our findings also showed moderately high odds ratios for these disorders and HIV risk behaviors.

A number of study limitations should be noted. The study cohort may not be generalizable to the US population of women living with HIV, and the cohort may not represent younger women or those who are not engaged in HIV care. In addition, space limitations prevent us from identifying associations between behavioral health disorders and factors such as income, race, education, and other demographic variables in the NCS-R comparator data. Another limitation is use of self-report for key study variables such as frequency and types of HIV risk behaviors. WMH-CIDI assessments were not available for the entire cohort, and this may have biased our results, especially those related to women who spoke only Spanish or those who were employed. Finally, we do not consider the effects of receiving treatment for behavioral health disorders or HIV/AIDS since these are the subject of a separate manuscript. Study strengths include the large number of participants, the multisite nature of the cohort, the diversity of geographic site locations, inclusion of a comparison population, and use of diagnostic interviews instead of screening tools.

The major implication of these results is the critical need for integrated health and behavioral health care and the importance of addressing the challenges currently faced in organizing and providing it to women living with HIV [58]. Studies have shown that screening HIV-positive adults and referring them to mental health and substance abuse treatment is largely ineffective [59], especially when psychiatric disorders and substance use disorders co-occur [60]. Moreover, the high rate of co-morbidity among HIV-positive women may make screening for discrete conditions an ineffective approach. Further, because women from diverse cultures experience and express behavioral health symptoms and role impairment differently [61, 62], screening and assessment must also be sensitive to African American, Hispanic/Latina, and other cultures [63, 64], as well as to the intersection of trauma with poor mental health and substance use in the lives of many HIV-positive women [65, 66].

The fact that women living with HIV have multiple co-occurring behavioral health disorders of high severity poses serious challenges to clinicians seeking to provide coordinated care. The term “health complexity” has been coined to refer to individuals with multiple co-morbid medical *and* behavioral health conditions whose care is complicated by personal and social factors such as housing, ethnicity, social support, and poverty, as well as by health system factors such as inadequate insurance coverage, gaps in specialty care, and segregated medical and behavioral health treatment [67, 68]. This characterization fits our study population especially well, and calls attention to specific challenges. One such challenge is a lack of evidence-based treatment options for people with co-occurring substance use disorders, mood disorders, and anxiety disorders. Research shows that treating only one of these disorders does not improve the others, and that when only one is treated, significant distress and disability typically remain [52, 69]. An alternative approach is concurrent treatment, in which separate therapies are delivered at the same time. For example, studies support combining cognitive behavioral therapy for substance use disorders with exposure therapy or cognitive restructuring for anxiety disorders [69]. Research also supports the use of integrated treatment approaches, where services that target multiple conditions simultaneously are delivered in a coordinated fashion. For example, a recent trial combining sertraline and naltrexone for treating co-occurring depression and alcohol dependence was effective in reducing depression and promoting alcohol abstinence [70]. However, development and evaluation of these models are in relatively early stages, meaning that we lack effective treatments for co-occurring behavioral health disorders [52], especially among women [71].

A second level of challenges involves integrating medical and behavioral health treatment in the face of multiple co-occurring conditions of each type along with personal and health system factors. Here again, there are promising models that can be applied to HIV-positive women with multi-morbidities. One is the collaborative care model in the primary care setting, where case managers or nurse practitioners work with primary care providers, mental health professionals, and substance use treatment specialists in a patient-centered, socioecologically focused approach that incorporates community and health system factors [72, 73]. Another model is integrated complex case management, which involves the longitudinal, relationship-based use of biopsychosocial and health system-based care plans that are complexity-focused, with few handoffs, and escalation of care as well as discharge based on clinical, functional, and cost outcome measurement [68]. Application of these models in the HIV field is relatively new, but they offer great promise [74–76], especially when designed to also address HIV-positive women’s key structural determinants including violence against women and economic insecurity [26].

In addition to the human costs of co-occurring HIV infection and behavioral health disorders, the financial costs are considerable. One recent analysis of patients in the Kaiser Permanente Northern California integrated health care system [77], reported that mean total healthcare costs were higher in HIV-positive patients who were diagnosed with psychiatric disorders and substance use disorders (per patient per year average of $32,881) compared to HIV-positive patients without these comorbidities (per patient per year average of $29,142), and that this difference remained significant after controlling for confounding individual characteristics. Taking this one step further, the strong association between having psychiatric disorders and substance use disorders and engaging in HIV risk behaviors increases the chances of viral transmission and creation of new infections, while lessening the positive potential of treatment as prevention [78], incurring additional societal costs and individual burdens.

We conclude that the development, testing, and diffusion of evidence-based, culturally sensitive, trauma-informed models of complex, integrated physical and behavioral health care should be a key public health strategy for HIV prevention and control. Also needed is further investigation of how race/ethnicity, income, education, poverty, and trauma affect the experience and expression of behavioral health disorders among women living with HIV. Finally, the impact of treatment for mental health and substance use disorders on the likelihood of engaging in HIV risk behaviors requires further exploration, in order to more effectively integrate behavioral health and antiretroviral therapies.
